# Induction Chemotherapy with FOLFIRINOX for Locally Advanced Pancreatic Cancer: A Simple Scoring System to Predict Effect and Prognosis

**DOI:** 10.1245/s10434-022-12569-y

**Published:** 2022-09-24

**Authors:** Masayuki Tanaka, Max Heckler, André L. Mihaljevic, Shigenori Ei, Ulla Klaiber, Ulrike Heger, Markus W. Büchler, Thilo Hackert

**Affiliations:** 1 Department of General, Visceral and Transplantation Surgery, University of Heidelberg, Heidelberg, Germany; 2 Department of Surgery, Keio University School of Medicine, Minato City, Japan; 3 Department of General and Visceral Surgery, University Hospital Ulm, Ulm, Germany; 4 Department of General Surgery, Division of Visceral Surgery, Medical University of Vienna, Vienna, Austria

## Abstract

**Background:**

Effective chemotherapy (CT*x*) protocols as induction treatment provide increasing opportunities for surgical resection of locally advanced pancreatic cancer (LAPC). Although improved survival after resection of LAPC with CT*x* has been reported for selected patients, reliable recommendations on the indication for conversion surgery after induction treatment are currently lacking. We investigated the factors predictive of prognosis in resected LAPC after FOLFIRINOX.

**Methods:**

Consecutive patients with LAPC undergoing curative resection after FOLFIRINOX between 2011 and 2018 were identified from a prospectively maintained database. Relevant clinical parameters and CT findings were examined. A scoring system was developed based on the ratio of hazard ratios for overall survival of all significant predictors.

**Results:**

A total of 62 patients with LAPC who underwent oncologic resection after FOLFIRINOX were analyzed. Tumor shrinkage, tumor density, and postchemotherapy CA19-9 serum levels were independently associated with overall survival (multivariate analysis: HR = 0.31, 0.17, and 0.18, respectively). One, two, and two points were allocated to these three factors in the proposed scoring system, respectively. The median overall survival of patients with a score from 0 to 2 was significantly shorter than that of patients with a score from 3 to 5 (22.1 months vs. 53.2 months, *P* < 0.001).

**Conclusions:**

Tumor density is a novel predictive marker for the prognosis of patients with resected LAPC after FOLFIRINOX. A simple scoring model incorporating tumor density, the tumor shrinkage rate, and CA 19-9 levels identifies patients with a low score, who may be candidates for additional treatment.

**Supplementary Information:**

The online version contains supplementary material available at 10.1245/s10434-022-12569-y.

Pancreatic cancer (PDAC) is the third-leading cause of death from cancer in western countries. At diagnosis, 80% of patients do not undergo resection, usually because of either metastatic disease (50–60%) or locally advanced disease (30%).^1^ Surgical resection offers the chance of a cure for patients with localized resectable disease. Even though systemic chemotherapy or chemoradiotherapy has been developed for patients with advanced disease, their overall survival without surgical resection remains low compared with that of patients eligible for upfront surgery.^2^

Locally advanced pancreatic cancer (LAPC) is defined by tumor contact or involvement with major vessels.^3^ These cases often are considered as initially unresectable. Effective chemotherapy, such as FOLFIRINOX (fluorouracil, leucovorin, irinotecan, and oxaliplatin), has an impact on the prognosis of patients with LAPC in the palliative setting.^4^ Although there remains considerable uncertainty regarding surgical indication for patients with initially unresectable LAPC, its beneficial effect on the survival of selected patients with a good response to preoperative treatment has been reported.^5–8^

To evaluate the effect of induction therapy for solid tumors, the guideline of Response Evaluation Criteria in Solid Tumours (RECIST1.1), which focuses on tumor burden, is widely applied.^9^ However, as the resectability of pancreatic cancer is defined by vascular involvement rather than tumor size, RECIST is not appropriate for the assessment of tumor response following preoperative treatment in the setting of LAPC.^10^ Although FDG PET/CT examinations were currently suggested as a radiologic marker for therapeutic effects of neoadjuvant treatment for PDAC, the change of CT density in the tumor after preoperative treatment that reflect tumor biology has not been reported as its marker.^11, 12^ In clinical practice, decisions regarding resection of LAPC after induction treatment have been made on a highly individual basis. No objective and reliable criteria identifying the patients most likely to benefit from surgery have been described.^13, 14^

Therefore, the purpose of this study was to evaluate the preoperative factors predictive of overall survival in patients who have undergone resection for LAPC after induction FOLFILINOX and to establish a predictive model with these factors, which may support decision-making for additional treatment in the surgical treatment of LAPC.

## Methods

### Study Design and Population

Consecutive patients who underwent induction chemotherapy with FOLFIRINOX as first-line treatment and subsequent oncologic resection for LAPC at primary diagnosis between February 2011 and June 2018 at the Department of Surgery, University of Heidelberg, were identified from a prospectively collected database and included in this study. LAPC was defined according to the NCCN guidelines.^3^ Patients with metastatic disease found incidentally at surgery were excluded. All data were collected and handled in compliance with the guidelines of the Institutional Ethics Committee after approval (Ethics Committee Approval No. S-011/2015). All patients provided written, informed consent.

Based on previous results, FOLFIRINOX was selected as first-line chemotherapy for the majority of patients with LAPC in our institute.^15, 16^ Although at least six cycles of FOLFIRINOX (as either an original or a modified regimen) were basically planned for patients with LAPC at first diagnosis, patients with fewer than six cycles (i.e., due to adverse events) also were included in this study. Patients with other protocols as second-line chemotherapy were excluded.

### Patient Surveillance During Chemotherapy and Imaging Investigation

From the time of initial diagnosis and commencement of induction chemotherapy with FOLFIRINOX, patients were reevaluated every 3 months during chemotherapy by computed tomography (CT), measurement of CA19-9 serum levels, and clinical examination.

The details of CT acquisition were described previously.^17^ Briefly, four phase images (nonenhanced, arterial, venous, and delayed phase) were acquired with a 3-mm slice thickness. Tumor conspicuity in the portal venous phase was greater than in any other phases.^18^ At baseline and follow-up examinations, the largest area of the primary tumor was measured in the portal venous phase on axial CT slices by manually tracing the tumor outline with a region of interest (ROI), and then the mean CT density of the area was evaluated.^19^ The CT density of a normal pancreas area without obstructive pancreatitis or atrophy was chosen as a control variable. The CT density attenuation in the tumor was calculated as (CT density of tumor area)/(CT density of normal pancreas area). The ROIs were drawn three times in the same CT slice, and the mean data of tumor size and that of CT density were regarded as the patient’s representative data. The tumor size response during chemotherapy was calculated as (postchemotherapy tumor size)/(prechemotherapy tumor size). In the same fashion, the response rate of tumor density was calculated as (postchemotherapy CT density attenuation)/(prechemotherapy CT density attenuation). The tumor regression of artery encasement, which should reflect resectability, was defined by any improvements in the length and degree of tumor contact along and around the superior mesenteric artery (SMA), celiac axis (CeA), and common hepatic artery (CHA), which are associated with the definition of resectability in the NCCN guidelines.^3^ Patients who were Lewis antigen-negative (CA19-9 < 5.0 U/mL) at primary diagnosis were excluded from the analyses of CA19-9.^20^

### Indications for Surgery and Surgical Approach

Explorative laparotomy was scheduled for those patients who fulfilled the following criteria after FOLFIRINOX: no tumor progression on imaging, declining (or stable) CA19-9 values and good general condition, and technically resectable disease (including the consideration of venous and arterial resections) after FOLFIRINOX. If progressive disease and/or a technically unresectable tumor were detected intraoperatively, explorative or palliative surgery was performed. Otherwise, patients underwent oncologic resection of the primary pancreatic tumor.

### Outcomes

The primary endpoint of this study was the assessment of predictive factors of overall survival in patients with oncologic resection after FOLFIRINOX, which were obtained preoperatively. Relevant patient characteristics and surgical outcomes were extracted from the institution’s prospectively maintained pancreatic database. Each continuous variable was divided by an individual cut-off value to convert it to a categorical variable.

### Statistical Analysis

Recurrence-free survival was calculated from the date of operation to the date of recurrence on imaging findings or clinical findings, the date of the last follow-up or death. Overall survival was defined as the time from the date of initiation of chemotherapy to the date of death from any cause. Survival times were compared using Kaplan–Meier survival analysis. Data were censored if the patients were alive at the time of the analysis or had been lost to follow-up. The optimal cutoff value of each continuous variable for overall survival was determined by a minimum *P*-value approach. Every possible cutoff point was examined by means of the log rank test. The value that provided the minimum *P* value was considered as the optimal cutoff point. A multivariate Cox regression model was utilized to determine significant predictors of prognosis. According to the hazard ratios (HRs) for overall survival of the significant predictors of the multivariable Cox regression, a score model was established. When the ratio of their HRs was translated into a score model, the minimal score among them was scaled down to 1, and the others were rounded off to the closest whole number. All statistical analyses were performed using *R* version 3.2.3 (*R* Project for Statistical Computing, Vienna, Austria), and *P* < 0.05 was considered to show a statistically significant difference.

## Results

### Demographics of the Study Cohorts and Surgical Outcomes

A total of 63 patients with LAPC who underwent oncological resection after FOLFIRINOX as the initial treatment were identified. Of the entire cohort of 63 patients, survival information was available for 62 patients (98%), and one patient was lost to follow-up. Thus, 62 patients were included in this study. Preoperative CA19-9 serum levels of 10 patients (16%) were not available (Table 1). The reasons for primary unresectability at diagnosis were arterial involvement in 48 (77%), venous involvement in 4 (6%), and both arterial and venous involvement in 10 patients (16%). Twenty-three patients (37%) underwent pancreaticoduodenectomy, 13 (21%) distal pancreatectomy, and 26 (42%) total pancreatectomy (Table 2). Portal vein resection was performed in 41 patients (66%), and major arteries (CeA/CHA/SMA) were resected in 12 patients (19%). *R*0 (at least 1 mm) and R0 (direct) resection were achieved in 19 patients (31%) and 45 patients (73%), respectively. Major morbidity (Clavien–Dindo grades lll–V) occurred in 26 patients (42%). Adjuvant chemotherapy following surgery was applied in 16 patients (26%).

**Table 1:** Patient demographics

Parameter	Patients
	*N* = 62
Age, years; median (range)	60 (36–83)
Male gender, *n* (%)	32 (52%)
Duration of preoperative FOLFIRINOX (mo); median (range)	3.8 (1.4–19.0)
RTx, *n* (%)	9 (15%)
*ASA score, n (%)*	
1	2 (3%)
2	35 (56%)
3	25 (40%)
Prechemotherapy CA19-9, U/mL; median (range)	968 (6–15000)
Postchemotherapy CA19-9, U/mL; median (range)	72 (6–3114)
*Reason for primary unresectability, n (%)*	
A	48 (77%)
PV	4 (6%)
A/PV	10 (16%)

*RTx* radiation therapy; *ASA* American Society of Anesthesiologists; *A* artery; *PV* portal vein

**Table 2:** Surgical outcomes

Parameter	Patients
	*N* = 62
*Type of pancreatectomy, n (%)*	
Partial pancreaticoduodenectomy	23 (37%)
Distal pancreatectomy	13 (21%)
Total pancreatectomy	26 (42%)
*Vascular resection, n (%)*	
Portal vein	41 (66%)
Artery (CeA, CHA, SMA)	12 (19%)
*Tumor characteristics*	
*T* stage, *n* (%)	
yp T0	1 (2%)
yp T1	3 (5%)
yp T2	17 (27%)
yp T3	39 (63%)
yp T4	2 (3%)
*N* stage, *n* (%)	
yp N0	27 (44%)
yp N1	22 (35%)
yp N2	13 (21%)
LNR^a^; median (range)	0.02 (0.00–0.36)
R0 (>1 mm) margin^a^, *n* (%)	19 (31%)
R0 (direct) margin^b^, *n* (%)	45 (73%)
Hospital stay, days; median (range)	14 (7–73)
Morbidity (Clavien–Dindo grade 3+), *n* (%)	26 (42%)
30-day mortality, *n* (%)	4 (6%)
*Adjuvant chemotherapy, n (%)*	
Yes	16 (26%)
No	41 (66%)
Unknown	5 (8%)
Disease free survival, months; median (range)	9 (1–72)
Overall survival, months; median (range)	34 (5–80)

^a^Minimum 1-mm margin

^b^More than 0-mm margin

*LNR* lymph node ratio, *CeA* celiac axis, *CHA* common hepatic artery, *SMA* superior mesenteric artery

### Defining Cutoff Values

The median disease-free survival was 9.0 months, and the median overall survival was 34.2 months. According to a minimum *P*-value approach by the log rank test to assess every possible cut-off value of each continuous variable for overall survival, the best cutoff point for the shrinkage rate of the tumor after chemotherapy was 0.5 (*P* = 0.017), that for the response rate of CT density attenuation in the tumor was 1.2 (*P* = 0.005), and that for the post-chemotherapy CA19-9 serum level was 100 (*P* = 0.004). The continuous variables were converted to dichotomous variables according to the defined cutoff values (Table 3).

**Table 3:** Optimal cutoff value of each potential predictor for overall survival

Variables	*P* value
Possible cutoff point	
*Shrinkage rate of primary tumor size after chemotherapy*	
0.3	0.986
0.4	0.177
0.5	0.017
0.6	0.247
0.7	0.118
0.8	0.076
0.9	0.283
1.0	1.000
*Response rate of CT value attenuation in primary tumor after chemotherapy*	
1.0	0.829
1.1	0.440
1.2	0.005
1.3	0.563
1.4	0.666
*Prechemotherapy CA19-9, U/mL*	
200	0.133
400	0.105
600	0.115
800	0.115
1000	0.146
*Postchemotherapy CA19-9, U/mL*	
50	0.015
100	0.004
150	0.012
200	0.012
250	0.012

### Univariate and Multivariate Survival Analysis

Univariate and multivariate analysis revealed that tumor shrinkage, tumor CT density attenuation, and postchemotherapy CA19-9 serum levels were significantly related to overall survival (univariate analysis: *P* = 0.017, *P* = 0.005, and *P* = 0.004; multivariate analysis: *P* = 0.030, *P* = 0.001, and *P* = 0.001, respectively) (Table 4). Tumor regression of arterial encasement did not predict prognosis (*P* = 0.086).

**Table 4:** Univariate and multivariate analysis of predictors of overall survival

Variables	Univariate analysis	Multivariate analysis		
	*P*	Hazard ratio	95% CI	*P*
*Shrinkage rate of primary tumor after chemotherapy*	0.017	0.31	0.108–0.892	0.030
≥0.5				
<0.5				
*Response rate of CT value attenuation in primary tumor after chemotherapy*	0.005	0.17	0.058–0.523	0.001
≥1.2				
<1.2				
*Postchemotherapy CA19-9, U/mL*	0.004	0.18	0.066–0.509	0.001
≥100				
<100				
*Regression of artery encasement*^a^	0.086	0.90	0.307–2.641	0.700
Yes				
N				

^a^Any effective response of artery (SMA, CHA, CA) encasement on CT was categorized as Yes

### Predictive Model

**Fig. 1:**
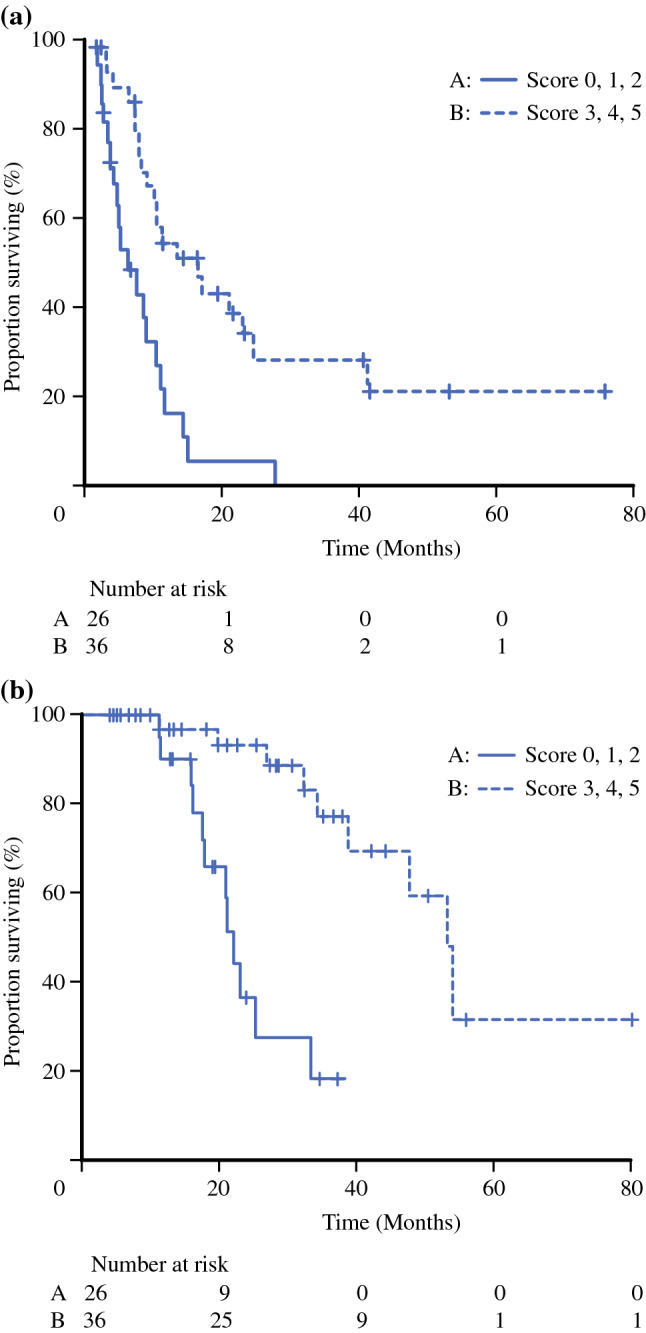
Recurrence-free survival (1–1) and overall survival (1–2) in patients who scored 0, 1, or 2 points (**a**) and those who scored 3, 4, or 5 points (**b**). There was a significant difference (*P* < 0.001 and *P* < 0.001, respectively)

In the multivariate analysis, the HRs for overall survival of the shrinkage rate of the tumor, the response rate of CT density attenuation, and the postchemotherapy CA19-9 serum level were 0.31, 0.17, and 0.18, respectively (Table 4). Accordingly, based on the effect size, a value of 1 (tumor shrinkage) or 2 (density and CA19-9) was assigned to each factor, resulting in a scoring system ranging from 0 to 5 points (Table 5). According to the scoring system, 26 patients (42%) had 0–2 points and 36 patients (58%) had 3–5 points. Type of pancreatectomy, *N* stage and LNR were significantly related to this system. However, duration of preoperative chemotherapy and adjuvant treatment were not associated with it (Supplementary Table 1). The median duration of follow-up was 21.2 (range, 4.2–80.3) months. There was a significant difference in recurrence-free survival and overall survival between the patients who scored 0 to 2 points and those who scored 3 to 5 points (*P* < 0.001 and *P* < 0.001, respectively). The median recurrence-free survival time was 5.0 (95% CI 3.1–9.0) months and 15.0 (95% CI 7.7–22.6) months, respectively. The median overall survival time was 22.1 (95% CI 17.6–33.4) months and 53.2 (95% CI 38.8–NA) months, respectively (Fig. 1).

**Table 5:** Score model for the prognosis of resected LAPC with induction FOLFIRINOX

Variables	Tumor shrinkage	Density improvement	Postchemotherapy CA19-9
HR	0.31	0.17	0.18
Score	≥ 0.5: 0	< 1.2: 0	≥ 100 U/mL: 0
	< 0.5: 1	≥ 1.2: 2	< 100 U/mL: 2

Score	*N* (%)
0	7 (11)
1	3 (10)
2	16 (26)
3	21 (34)
4	4 (6)
5	11 (18)

## Discussion

In this study, the shrinkage rate of the primary tumor, the response rate of CT density attenuation of the tumor, and postchemotherapy CA19-9 serum levels were independent predictors of survival in patients with resected LAPC after preoperative treatment with FOLFIRINOX. The predictive model with these 3 factors stratified patients who benefit from oncologic resection.

Patients with LAPC, representing an advanced stage of the disease, are either considered impossible to resect with negative resection margins or suspected to have undetected metastases or micrometastases.^21^ With the advent of effective CT*x* protocols, such as FOLFIRINOX, this paradigm is currently shifting, and some patients initially deemed unresectable can proceed to subsequent surgical resection.^5, 6, 22^ Identifying those patients who would benefit from conversion surgery is an unmet demand in multidisciplinary PDAC treatment. Evidence-based guidelines giving clear recommendations for secondary resection are lacking, due to lack of reliable response evaluation or validated prediction models to stratify patients. Recent developments in chemotherapy may provide more opportunities for potentially curative resection in carefully selected patients with LAPC.^14, 23^ In our institution, the eligibility criteria for surgical exploration are lack of tumor progression and technically resectable disease on CT following induction treatment. Because the application of these rules results in a wide range of survival (survival time after surgery: 0.8–71.8 months), no beneficial surgery should be avoided whenever possible.

Predictive factors for the prognosis of LAPC patients following induction chemotherapy and resection have been debated controversially in the past decade.^24^ As the change in the apparent radiographic extent of the tumor is rarely visible after chemotherapy for pancreatic cancer, radiologic characteristics are considered unsuitable for decision making.^25, 26^ However, in cases of radiologic evidence of regression of tumor size and tumor-vessel contact conversion surgery may be indicated and related with a survival advantage.^27^ With regard to biomarkers, a decline in serum CA19-9 levels after induction treatment was shown to be associated with prolonged survival in patients undergoing secondary resection of LAPC.^28^ With improved operative safety for pancreatic resection and more effective chemotherapeutic regimens, prediction models incorporating available preoperative parameters are desirable to stratify patients for further treatment.

In this study, among CT findings and tumor markers obtained preoperatively, radiologic tumor size, tumor CT density and postchemotherapy CA19-9 serum levels were identified as prognostic relevant factors to stratify the LAPC patients for conversion surgery after preoperative FOLFIRINOX in terms of overall survival. As this study was performed in a patient population with LAPC undergoing resection after fulfilling our criteria for conversion surgery, these three factors might be associated with the effect of chemotherapy on tumor regression. Among these factors, only the shrinkage rate of the tumor is included in the RECIST guidelines.^9^ CT contrast enhancement of the tumor reflects the status of microvascular formation and its biology, suggesting the prognosis.^12, 29^ The CT density of well-differentiated adenocarcinoma is higher than that of moderately or poorly differentiated adenocarcinoma. However, the response rate of tumor density attenuation on CT has not been reported to provide useful information on the prognosis of LAPC patients yet. PDAC is characterized by an extensive and dense desmoplastic or fibrotic stroma.^25, 30^ Thus, its response rate on CT would reflect extensive desmoplastic reaction and differentiation between tumor cells and fibrosis after chemotherapy. Besides imaging findings, more attention has currently been paid to the CA19-9 serum level as a predictive marker of the effect of chemotherapy for pancreatic cancer.^28, 31, 32^ Unexpectedly, the tumor vascular contact around SMA, CeA, and CHA were rarely changed after chemotherapy and were not considered predictors.^27^

According to the univariate and multivariate analyses, we established a score system with the weighted values based on the HRs for the prognosis of resected LAPC. Generally, a scoring system should depend on its intended application.^33^ Here, the clinical matter in the setting of initially unresectable LAPC with resection following induction chemotherapy is whether a series of treatments is sufficient. Additional treatment before and after resection, such as extension of preoperative treatment and performing adjuvant therapy, should be adopted for the patients with worse prognosis. Classification into two groups by setting a boundary between the score results of 2 and 3 might be optimal for determining whether additional treatment is needed.

Some limitations need to be addressed. First, as patients fulfilling our surgical criteria for LAPC but then not undergoing resection due to poor performance status and patient refusal were not included, the survival benefit from oncologic resection could not be assessed. The true impact of the score model on decision-making for resection requires reevaluation in further studies, preferably within a multicenter, prospective study design. Second, dose modification of FOLFIRINOX and the timing of the prechemotherapy CA19-9 test (e.g., before or after biliary drainage) may have contributed to the risk of bias. Third, FDG PET/CT examinations were not performed in this study, which might be associated with therapeutic effects of neoadjuvant treatment for PDAC.^11^ Lastly, it is unclear whether this model is applicable to all patients treated with different chemotherapy regimens, such as gemcitabine-based chemotherapy. To avoid the heterogeneity of morphologic response by various agents, we focused exclusively on FOLFIRINOX.

## Conclusions

In the context of patient selection for surgical resection after induction therapy, predictive factors for the prognosis of patients with resected LAPC after FOLFIRINOX could be identified and a simple model containing three factors was established. The major advantage of this score model is the preoperative availability of all parameters after completion of neoadjuvant therapy and before intended surgical resection. Patients with a higher score are definitive candidates for resection and may achieve excellent survival times. However, patients with a low score may not necessarily have to be excluded from surgical exploration, yet they may be candidates for additional treatment. This should be addressed in larger, prospective studies in the future.

## Supplementary Information

Below is the link to the electronic supplementary material.

Supplementary file1 (DOCX 15 kb)
