# Supplementary material for: Induction Chemotherapy with FOLFIRINOX for Locally Advanced Pancreatic Cancer: A Simple Scoring System to Predict Effect and Prognosis
Source: Ann Surg Oncol. 2022 Sep 24;30(4):2401–8. doi: 10.1245/s10434-022-12569-y (PMC10027766; doi:10.1245/s10434-022-12569-y)
Supplement: Supplementary file 1 — Supplementary file1 (DOCX 15 kb) [file 10434_2022_12569_MOESM1_ESM.docx]

**Supplementary Table 1** Patient characteristics and outcomes in the scoring system

| **Parameter** | | | **0-2 scores** | **3-5 scores** |  |
| --- | --- | --- | --- | --- | --- |
|  |  |  | **N = 26** | **N = 36** | ***P*** |
| Age, years; median (range) | | | 61 (36–83) | 59 (43–76) | 1.000 |
| Male gender, n (%) | |  | 15 (58%) | 17 (47%) | 0.451 |
| Duration of preoperative FOLFIRINOX, months; median (range) | | | 3.1 (1.4–19.0) | 4.1 (1.4–11.1) | 0.317 |
| RTx, n (%) | |  | 4 (15%) | 5 (14%) | 1.000 |
| ASA score, n (%) | |  |  |  | 0.800 |
|  |  | 1 | 1 (4%) | 1 (3%) |  |
|  |  | 2 | 16 (62%) | 19 (53%) |  |
|  |  | 3 | 9 (35%) | 16 (44%) |  |
| Reason for primary unresectabiliy, n (%) | | |  |  | 0.810 |
|  |  | A | 19 (73%) | 29 (81%) |  |
|  |  | PV | 2 (8%) | 2 (6%) |  |
|  |  | A/PV | 5 (19%) | 5 (14%) |  |
| Type of pancreatectomy, n (%) | | |  |  | 0.021 |
|  |  | Partial pancreaticoduodenectomy | 5 (19%) | 18 (50%) |  |
|  |  | Distal pancreatectomy | 5 (19%) | 8 (22%) |  |
|  |  | Total pancreatectomy | 16 (62%) | 10 (28%) |  |
| Vascular resection, n (%) | | |  |  |  |
|  |  | Portal vein | 18 (69%) | 23 (64%) | 0.788 |
|  |  | Artery (CeA, CHA, SMA) | 3 (19%) | 9 (25%) | 0.214 |
| Tumor characteristics | | |  |  |  |
|  | T stage, n (%) | |  |  | 0.273 |
|  |  | yp T0 | 0 (0%) | 1 (3%) |  |
|  |  | yp T1 | 1 (4%) | 2 (6%) |  |
|  |  | yp T2 | 4 (15%) | 13 (36%) |  |
|  |  | yp T3 | 20 (77%) | 19 (53%) |  |
|  |  | yp T4 | 1 (4%) | 1 (3%) |  |
|  | N stage, n (%) | |  |  | 0.004 |
|  |  | yp N0 | 5 (19%) | 22 (61%) |  |
|  |  | yp N1 | 13 (50%) | 9 (25%) |  |
|  |  | yp N2 | 8 (31%) | 5 (14%) |  |
|  | LNR*; median (range) | | 0.05 (0.00–0.36) | 0.00 (0.00–0.30) | 0.004 |
|  | R0 (>1 mm) margin*, n (%) | | 5 (19%) | 14 (39%) | 0.098 |
|  | R0 (direct) margin^†^, n (%) | | 18 (69%) | 27 (75%) | 0.774 |
| Hospital stay, days; median (range) | | | 14 (7–73) | 14 (7–43) | 0.715 |
| Morbidity (Clavien–Dindo grade 3+), n (%) | | | 13 (50%) | 13 (36%) | 0.307 |
| 30-day mortality, n (%) | | | 3 (12%) | 1 (3%) | 0.300 |
| Adjuvant chemotherapy, n (%) | | |  |  | 1.000 |
|  |  | Yes | 7 (27%) | 9 (25%) |  |
|  |  | No | 17 (65%) | 24 (67%) |  |
|  |  | Unknown | 2 (8%) | 3 (8%) |  |

*Minimum 1-mm margin

†More than 0-mm margin

*RTx* radiation therapy; *ASA* American Society of Anesthesiologists; *A* artery; *PV* portal vein; *LNR* lymph node ratio; CeA celiac axis; *CHA* common hepatic artery; *SMA* superior mesenteric artery
